# Non-accidental broom sticks injury as a cause of post-neonatal tetanus

**DOI:** 10.11604/pamj.2019.34.143.20606

**Published:** 2019-11-13

**Authors:** Eyong Komomo, Torty Chimaeze, Ekanem Emmanuel, Asindi Asindi

**Affiliations:** 1 Department of Paediatrics, University of Calabar Teaching Hospital, Calabar, Nigeria

**Keywords:** Tetanus, broom stick injury, Nigeria

## Abstract

**Introduction:**

Non accidental injury sustained following deliberate self-harm or inflicted by parents or caregivers for disciplinary, traditional and therapeutic measures have grave consequences including exposing the incompletely child to post neonatal tetanus. This contributes to the continuing high incidence of post neonatal tetanus in developing countries.

**Methods:**

A 12 year retrospective review of all children admitted into the children’s ward of the University of Calabar Teaching Hospital with diagnosis of post neonatal tetanus was carried out. The demographic characteristics of the children were documented. Immunization status, possible portal of entry and outcome status were also recorded. Data obtained was analysed using SPSS version 22.

**Results:**

There was a male preponderance of 70.5% amongst the 44 children with post neonatal tetanus. Non-accidental injuries accounted for 20(45.5%). Broom stick injury sustained during corporal punishment was responsible for 60% of tetanus from the non-accidental injury group. Low socio economic class and Inadequate or no immunizations are major risk factors for tetanus infection.

**Conclusion:**

Non accidental injuries inflicted on children as disciplinary measures accounted for about half of children with post neonatal tetanus. Broom stick injury was a major contributory factor. Lack of immunization and low social class remains major risk factors for post neonatal tetanus. Post neonatal tetanus presents another reason for sustained campaign against physical abuse of children.

## Introduction

Non-accidental injury results from a complex pathological interaction between the individual and the community [1]. It is a leading cause of death among children and young adults [2]. Over 875 000 children ≤ 18 years of age die annually in the world as a result of injuries, mostly in low- and middle-income countries (LMIC), where injuries account for 13% of the total burden of morbidity among children ≤ 15 years of age [3,4]. Non-accidental or intentional childhood injury includes injuries sustained by a child as result of deliberate self-harm or injuries deliberately inflicted by parents or caregivers for disciplinary, traditional and therapeutic interventions purposes [4,5]. These injuries may lead to physical, emotional and psychological harm to the child [4,5]. The practice of inflicting injuries on children intentionally is common in the developing world but a complex interplay of factors continues to downplay its recognition and reportage [4-6]. Post neonatal tetanus is a non-communicable disease of major public health concern in Nigeria and other developing countries [7]. The incidence has remained unacceptably high in developing countries despite being a vaccine-preventable disease [8,9] with the existence of the childhood immunization programs for over four decades [10]. The contributory factors to this continued high incidence of tetanus include lack of, or incomplete immunization among other reasons [2]. Previous studies have shown that most cases of post neonatal tetanus are due to accidental injuries occurring in non-immunized individual but we are presenting in this study, post neonatal tetanus from non-accidental broom stick and other injuries inflicted on children by parents or caregivers.

## Methods

The study was a retrospective study of all children admitted into the Children’s ward of the University of Calabar Teaching Hospital with diagnosis of post neonatal tetanus between September 2008 and October 2017. The demographic characteristics of the children including the age, sex, ethnic group and religion. Social class of the children using the Oyedeji classification [11] which utilizes the educational background and occupation of parents. Possible portal of entry of the Clostridium tetanus and outcome status were documented. Data analysis was done using Microsoft Excel statistical package supplemented with Statistical Package for Social Sciences (SPSS) version 22. Mean, standard deviation and other parameters were generated as necessary for continuous data. Means of continuous variables were compared using the Student t- test and categorical variables using Chi-square test. Level of significance set at p< 0.05.

## Results

Forty-four children with post neonatal tetanus were seen in our facility within the period under review. Of these number, thirty-one (70.5%) were males while thirteen (29.5%) were female giving a male female ratio of 2.5:1). The age group 5-10 has the highest of children (22; 14 males and 8 females) with post neonatal tetanus; this is closely followed by the age group 11-15 years (18; 13 males and 5 females). The least incidence was amongst children aged 5 years and above 15 years with two cases each Forty (90.9%) of the children in the study are from the low social class while the other four are from the middle social class Accidental injuries accounted for 21 (47.7%), this is closely followed by non-accidental injuries in 20 children representing 45.5% (Table 1). Broom stick injuries accounted for 60% of tetanus from non-accidental injuries. Of the 44 children seen with post neonatal tetanus in the study, only 9 (20.5%) received complete immunization against tetanus. Twenty (45.5%) of the children had incomplete immunization while 15 (34.1%) of them had no immunization. Thirty-four (77.3%) of the 44 children with post neonatal tetanus in the study survived. Of the 10 children who died from the disease eight were not immunized. There is a significant relationship between immunization against tetanus and survival (P<0.05) (Table 2).

**Table 1:** Aetiopathogenesis of post neonatal tetanus

Type of injury	frequency	percentage
**Accidental**	21	47.7
**Non accidental**	20	45.4
Broom stick injuries	12	
Others	8	
**Unknown**	3	6.8
Total	44	100

**Table 2:** Comparison of immunization status and out come

Immunization status	Outcome	
**Total**	**Survived**	**Died**
**Yes** (9)	7	2
**No** (35)	27	8
Total (44)	34	10
		p < 0.05

## Discussion

Non-accidental childhood injuries sustained as a result of deliberate self-harm or inflicted by parents or caregivers for disciplinary, traditional and therapeutic interventions purposes may lead to physical, emotional and psychological harm to the child [4-6]. In this 12 year review of post neonatal tetanus cases in the University of Calabar Teaching Hospital Nigeria, twenty (45.5%) of the children developed tetanus as a result of injuries inflicted on them by either care gives or parents as disciplinary measures. It is important to note that 60% of tetanus from non- accidental injuries in this study were due to broom stick injuries. This confirms the results of earlier studies in Nigeria that broom stick constitute a significant cause of post neonatal tetanus [12-15]. Broom sticks are used in most homes in Nigeria to sweep the floor which is the natural habitat of clostridium tetanus and therefore at risk of harbouring the organism. The practice of flogging children with broom by parents or care givers and teachers in schools as a form of corporal punishment for erring pupils [16] exist with attendant risk of penetrating injuries to the child that may lead to tetanus.

Immunization is key in preventing post neonatal tetanus infection as evidence abounds in literature that lack or incomplete immunization is the major risk factor for tetanus infection [17-20]. In this study, approximately 80% of children who had tetanus had inadequate or no immunization. The DPT3 completion rate in Nigeria within the period under review ranged between 40-60% [21] implying a large number of Nigerian children are either inadequately or not immunized against tetanus or therefore prone to post neonatal tetanus. The dominance of school age children with post neonatal tetanus in the study shows the need to administer booster doses of tetanus toxoid to all children as part of the national immunization programme since immunity is known to wane during school age years in those who previously received the vaccine at infancy without follow up booster doses [22]. Studies in parts of South Eastern Africa show that tetanus seroprotection was lower among children 5-14 years when compared to those aged 1-4 years demonstrating waning immunity with age [22].

This study demonstrated that the social class of parents plays an important role in the likelihood of having tetanus; over 90% of the children in this study were from the low social class. Expectedly, no child from the high social class had post neonatal tetanus. Children in the low social class are the less likely to have complete immunization and are more likely involved in high risk behaviours that makes them vulnerable to tetanus infection. It would appear that corporal punishment is also more common among this group.

## Conclusion

Non accidental injuries inflicted on children by care gives or parents as disciplinary measures accounted for about half of children with post neonatal tetanus. Broom stick injury was a major contributory factor. Incomplete or no immunization and low social class still remains major risk factors for post neonatal tetanus. There need for sustained campaign against physical abuse of children and complete immunization of every child to prevent post neonatal tetanus.

### What is known about this topic

- Accidental injuries causes tetanus;
- Unimmunized children are at a high risk of tetanus infection;
- Decline in immunity occurs after the age of five years.

### What this study adds

- Non accidental broom stick injury sustained during corporal punishment a major contributory factor to post neonatal tetanus;
- Broom stick harbors clostridium tetanus since use in sweeping the floor (the natural habitat of clostridium tetanus) in sub Saharan Africa.

## Competing interests

The authors declare no competing interests.
